# Keratoconus and inflammatory bowel disease: mendelian randomization

**DOI:** 10.3389/fgene.2024.1331751

**Published:** 2024-07-19

**Authors:** Yiheng Jin, Yuanfeng Wang, Xu Qiu, Jiao Liu, Shugen Qu

**Affiliations:** ^1^ School of Public Health, Zhejiang Provincial Key Laboratory of Watershed Science and Health, Wenzhou Medical University, Wenzhou, China; ^2^ South Zhejiang Institute of Radiation Medicine and Nuclear Technology, Wenzhou, China

**Keywords:** keratoconus, IBD, inflammatory, mendelian randomization (MR), UC

## Abstract

**Background:**

Keratoconus is a diseased corneal dilation of unknown etiology. Studies have shown that inflammation may play a role in keratoconus. Inflammatory enteritis (IBD), including ulcerative colitis (UC), is a chronic, systemic inflammatory disease. We used Mendelian randomization to assess the causal relationship among IBD, UC and keratoconus.

**Methods:**

The instrumental variable of IBD and UC was selected, the information of the instrumental variable in keratoconus outcome was extracted, and the causal relationship was assessed by the inverse variance weighted method by primary analysis, and its relevant sensitivity analysis.

**Results:**

A causal relationship between IBD and keratoconus was observed significantly (*P* = 0.017, OR = 1.21, 95% CI = 1.03–1.41), and same as to UC and keratoconus (*P* = 0.038, OR = 1.25, 95% CI = 1.01–1.54).

**Conclusion:**

IBD may play a causal role in the development of keratoconus, but the mechanism needs to be further elucidated.

## 1 Introduction

Keratoconus is an eye disease characterized by the thinning of the center of the cornea and protruding forward and conical. It often causes highly irregular myopia, astigmatism, and varying degrees of vision damage (Santodomingo-Rubido et al., 2022). Traditionally, keratoconus is considered a non-inflammatory disease (Krachmer et al., 1984; Rabinowitz, 1998). However, recent studies have found that some inflammatory molecules are overexpressed in the tears of patients with keratoconus: this suggests that there is an inflammatory component in keratoconus pathogenesis. Many clinical perspective examines the evidence and implications of numerous inflammatory processes that have been recognized in the tears of keratoconus patients, as well as some inflammation relevant differences found in the keratoconus cornea, it was significantly increased tear expression of MMP-13, IL-6, IL-17, TNF-α and TNF-β was evident in keratoconus tears. The roles of inflammation in corneal trauma attributed to eye rubbing and/or contact lens wear are examined as is the significance of atopy, allergic disease, dry eye disease, degradative enzyme activity, wound healing, reduced anti-inflammatory capacity, and ultraviolet irradiation (Lema and Durán, 2005; Lema et al., 2009; Galvis et al., 2015b; McMonnies, 2015; Wisse et al., 2015). Inflammatory bowel disease (IBD), which encompasses Crohn’s disease and ulcerative colitis (UC), is a chronic inflammatory disease of the intestine of unknown etiology (Peyrin-Biroulet et al., 2008; Sahoo et al., 2023). Some eye complications are common in IBD (Knox et al., 1984). The possible association of IBD and keratoconus had not been established. However, a cross-sectional study had suggested that individuals with IBD may be at an elevated risk of developing keratoconus (Tréchot et al., 2015). This implies that there may be some common pathological pathways between IBD and keratoconus, and that IBD and keratoconus shared some common inflammatory activities, resulting in a higher incidence of keratoconus in IBD patient.

Mendelian randomization (MR) is an epidemiological data analysis technique that leverages genetic variations robustly associated with exposure factors to serve as instrumental variables. This approach is particularly adept at evaluating the causal relationships between exposure factors and health outcomes. A significant advantage of MR is its ability to mitigate biases stemming from confounding factors and reverse causality, thereby enhancing the validity of etiological inferences (Lawlor et al., 2008; Emdin et al., 2017).

In this study, we used a two-sample MR design to identify potential associations and causal links between IBD and ulcerative colitis with keratoconus as outcomes (Crohn’s disease was not included in the study because no data was available) provided data support for corresponding scientific research.

## 2 Materials and methods

### 2.1 Data sources

Genome-wide association studies (GWAS) of keratoconus, IBD and UC are from the GWAS database (http://gwas-api.mrcieu.ac.uk/, accessed on 6 August 2023). Because it is publicly available data, no additional ethical approval is required. The data for IBD, UC and keratoconus are also from GWAS datasets. The details of the included GWAS are shown in Table 1.

**TABLE 1:** The database source for MR analysis.

Exposure/outcome	GWAS ID	Sample size	Number of SNPs	Population	Consortium	Sex	Year
Inflammatory bowel disease	ieu-a-292	75,000	14,378	European	IIBDGC	Males and females	2012
Ulcerative colitis	ieu-a-32	27,432	12,255,197	European	IIBDGC	Males and females	2015
Keratoconus	finn-b-H7_CORNEALDEFORM	2,09,287	16,380,407	European	NA	Males and females	2021

IIBDGC, International Inflammatory Bowel Disease Genetics Consortium; NA, no data.

### 2.2 Selection of instrumental variables

The selection of all instrumental variables (IVs), that are SNPs must meet the following three conditions (Gagliano Taliun and Evans, 2021): 1) There is a strong association between IVs and exposure (Rabinowitz, 1998). 2) IVs are independent of confounders affecting exposure and outcome (Krachmer et al., 1984). 3) IVs are not directly associated with outcome, and they can only exert effects on outcome by exposure pathway.

At the beginning, we extracted SNPs which are strongly associated with inflammatory enteritis and ulcerative colitis from already published data, we use *P* < 5 × 10^−8^ as the primary screening condition. To ensure that the exposure tools we use are independent and random, we excluded SNPs which are in linkage disequilibrium (LD) (r2 < 0.001, clumping window = 10,000 kb). We then extracted the relevant exposure instrumental variables in the GWAS data of keratoconus, and we excluded Palindromic SNPs. The remaining instrumental variables are considered instrumental variables.

### 2.3 Statistical analysis

To verify the causal association between the exposures and outcome. Several MR methods were used, including inverse variance weighted (IVW), MR-Egger, Weighted median, Simple mode, and Weighted mode. In international MR analysis, two methods (IVW and MR-Egger) are often used as basic MR methods. As the primary MR analysis method, IVW evaluates the association strength between instrumental variables and the outcome through regression analysis. This method assumes homogeneity in the associations of all instrumental variables with the outcome, that is, no horizontal pleiotropy exists. The IVW method is not sensitive to the strength of individual instrumental variables but is susceptible to outliers. MR-Egger extends IVW by allowing the assessment of potential horizontal pleiotropy, where instrumental variables may affect the outcome through pathways unrelated to the exposure. MR-Egger regression detects such pleiotropy by introducing an intercept term, and the significance of this intercept can be used to assess the presence of pleiotropy. Weighted Median method as a robust MR method, which is not sensitive to outliers. It uses the median effect estimate to reduce the impact of outliers, enhancing the robustness of the analysis. Simple Mode and Weighted Mode methods, aim to improve the efficiency of the analysis by selecting SNPs most closely associated with the instrumental variables, may be sensitive to pleiotropy. We used IVW as the primary method for MR analysis. When the *P* of the IVW method is less than 0.05, the results are considered meaningful. At the same time, it is also necessary to ensure that the b value direction of the IVW, MR-egger and Weighed media methods is consistent.

### 2.4 Sensitivity analysis

Heterogeneity was detected by Cochran’s Q test and pleiotropy was assessed by MR-Egger regression (Bowden et al., 2015; Greco et al., 2015). MR-presso test rejection anomalous SNP was used to detect and correct the level pleiotropy (Verbanck et al., 2018). Finally, the results were visualized with a one-by-one culling test, forest plot, scatter plot and funnel plot.

## 3 Result

### 3.1 Selection of instrumental variables between IBD and keratoconus

**FIGURE 1:**
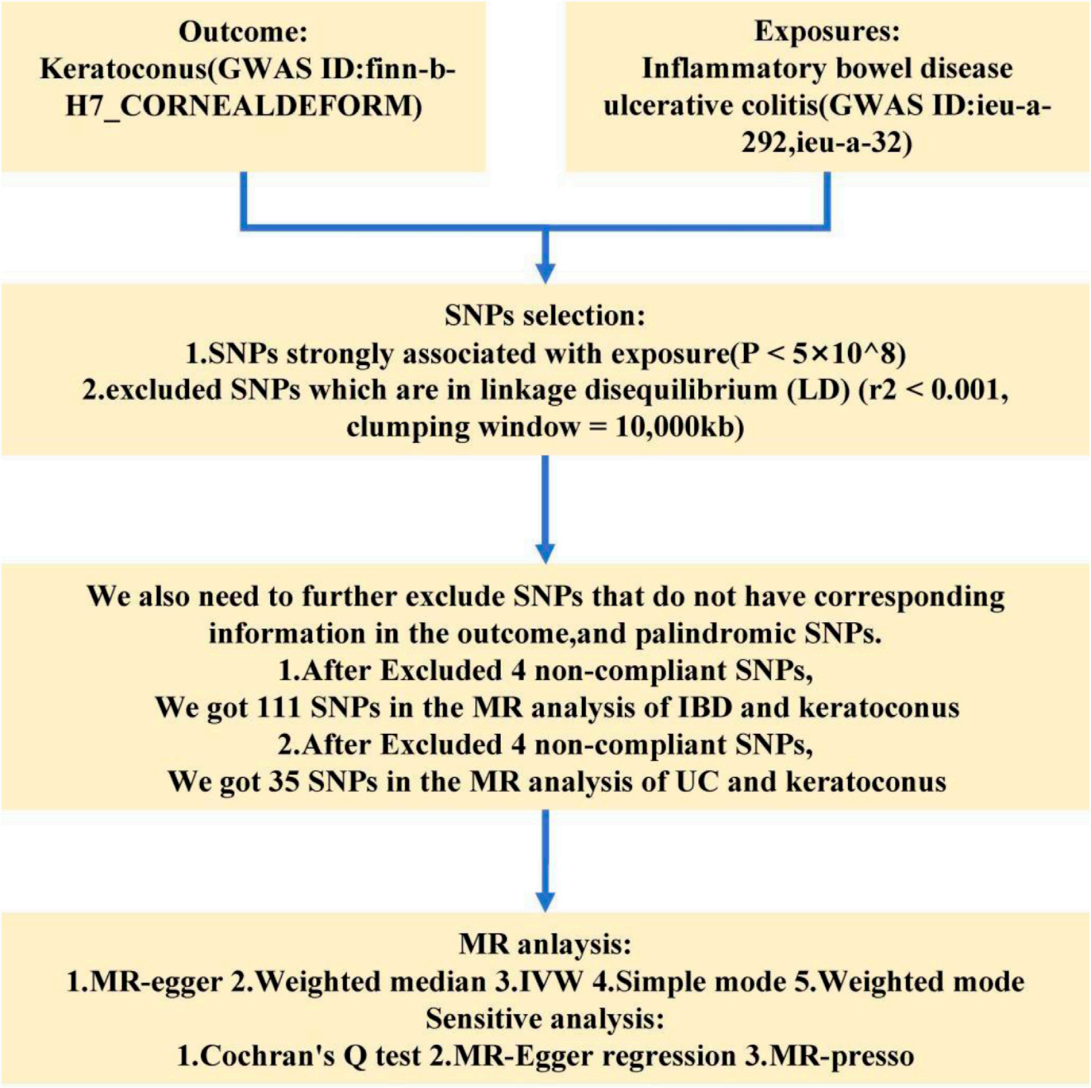
The flow chart of this study.

In the MR analysis of IBD and keratoconus, we extracted instrument SNPs with strong correlation properties (*P* < 0.05). At the same time, we had also removed the LD SNPs. We got 114 SNPs. Next, when extracting the information of instrumental in the outcome, We found 1 SNPs that were excluded due to no corresponding outcomes. When harmonizing exposure data and outcome data, there were 2 SNPs (rs17835641, rs1991866) excluded because they were palindromic SNPs. We ended up with 111 SNPs for MR analysis (Supplementary Tables S1, S2). Following a similar approach for the MR analysis of ulcerative colitis (UC) and keratoconus, we initially identified 39 SNPs, from which we excluded three SNPs lacking outcome information and one palindromic SNP (rs9891174) (Supplementary Tables S3, S4). We ended up with 35 SNPs for MR analysis. Figure 1 showed the flow chart of this study.

### 3.2 MR analysis results of IBD and keratoconus

In the MR analysis results of IBD and keratoconus, the risk used by IVW model showed that IBD was positively correlated with keratoconus (*P* = 0.017, OR = 1.21, 95% CI = 1.03–1.41). While in UC and keratoconus, the results of IVW model showed that UC was positively correlated with keratoconus (*P* = 0.038, OR = 1.25, 95% CI = 1.01–1.54). All data was shown in Table 2.

**TABLE 2:** The results of MR analyis between IBD and UC with keratoconus.

Exposure/outcome	Nsnp	Method	OR (95% CI)	*P*	se
IBD/keratoconus	111	MR-egger	1.13 (0.80–1.66)	0.531	0.20
		Weighted median	1.16 (0.92–1.47)	0.205	0.12
		IVW	1.21 (1.03–1.41)	0.017	0.08
		Simple mode	1.22 (0.70–2.13)	0.476	0.28
		Weighted mode	1.22 (0.84–1.78)	0.292	0.19
UC/keratoconus	35	MR-egger	1.72 (0.94–3.17)	0.089	0.31
		Weighted median	1.19 (0.93–1.52)	0.174	0.13
		IVW	1.25 (1.01–1.54)	0.038	0.11
		Simple mode	1.01 (0.62–1.63)	0.974	0.24
		Weighted mode	1.23 (0.89–1.71)	0.215	0.17

### 3.3 Sensitive analysis between horizontal pleiotropy and heterogeneity

To assess the pleiotropy and heterogeneity, we performed sensitivity analyses. The results of the sensitivity analysis were been shown in Table 3. There was no evidence to support pleiotropy of SNPs in the MR-Egger regression analysis (*P* = 0.732 > 0.05, *P* = 0.276 > 0.05). When testing for heterogeneity using Cochran’s Q, it was found that the tool SNPs in IBD and keratoconus analysis were not heterogeneous (*P* = 0.324 > 0.05), while SNPs in UC and keratoconus analysis were significantly heterogeneous (*P* = 0.009 < 0.05). It should be noted that when using the MR-PRESSO test, since the test requires simulation, and the large number of values of the dependent variable, the results obtained during each regression test are different, and the data was the average of multiple results. Next, in the analysis of IBD and keratoconus, instrumental SNPs did not show horizontal pleiotropy (*P* = 0.338 > 0.05) and did not exhibit outlier SNPs. But in the analysis of UC and keratoconus, instrumental SNPs table first came out with significant horizontal pleiotropism (*P* = 0.005 < 0.05), and there was a chance of outlier SNP (rs6062496). Owing to the stochastic nature of the MR-PRESSO test in this context, we chose not to exclude any outlier SNPs from the initial MR analysis.

**TABLE 3:** The results of sensitive analysis.

Exposure/outcome	Horizontal pleiotropy					Heterogeneity	
	MR-Egger regression			MR-PRESSO		Cochran’s Q	*P* value
	Egger intercept	SE	*P* value	Global test *P* value	Outliers		
IBD/keratoconus	0.008	0.022	0.732	0.338*	Null*	116.23	0.324
UC/keratoconus	−0.064	0.058	0.276	0.005*	rs6062496*	56.33	0.009

**FIGURE 2:**
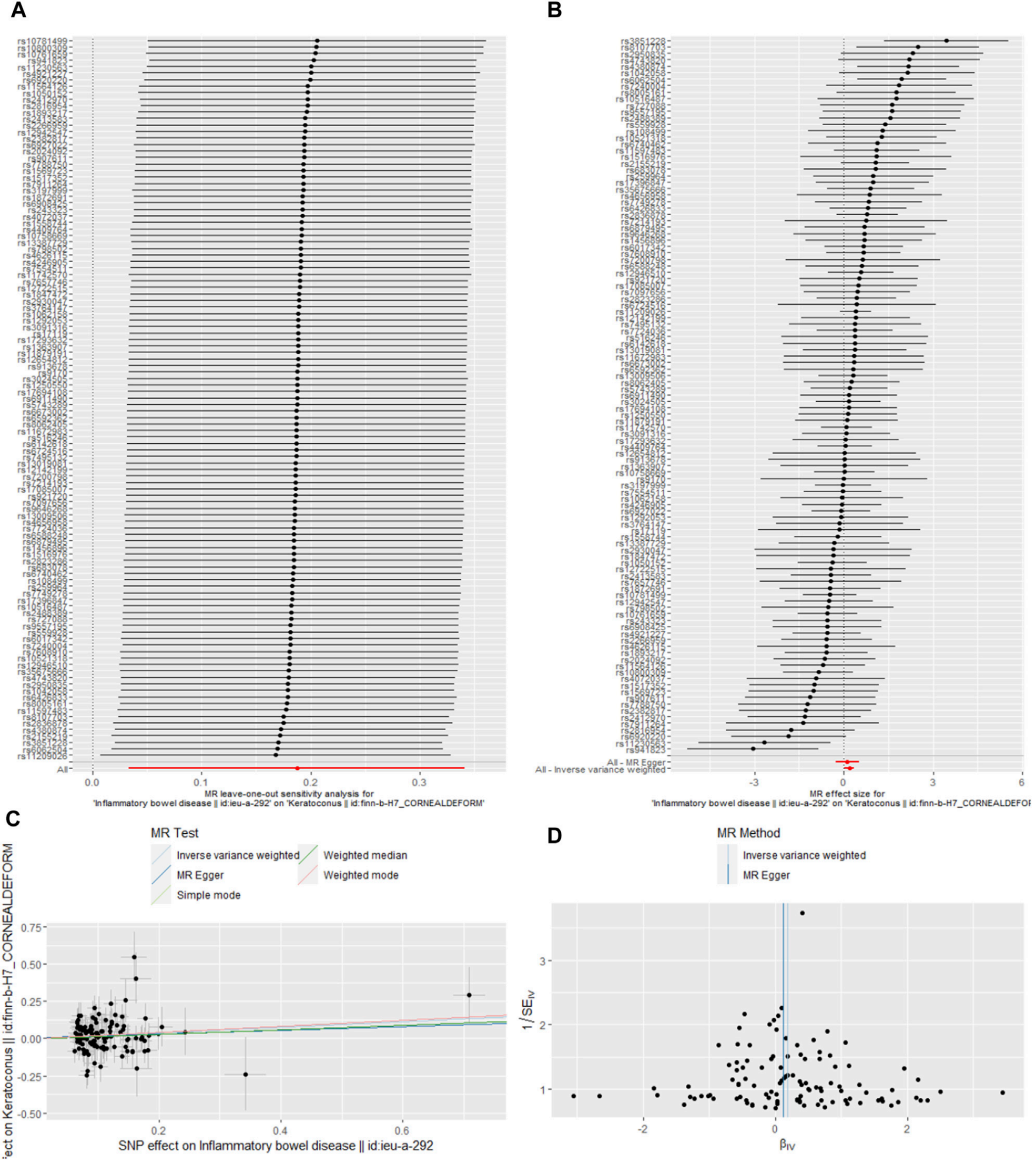
Sensitivity analysis of the causal effect of IBD on keratoconus risk. **(A)** leave-one-out plot, **(B)** forest plot, **(C)** scatter plot, **(D)** funnel plot.

**FIGURE 3:**
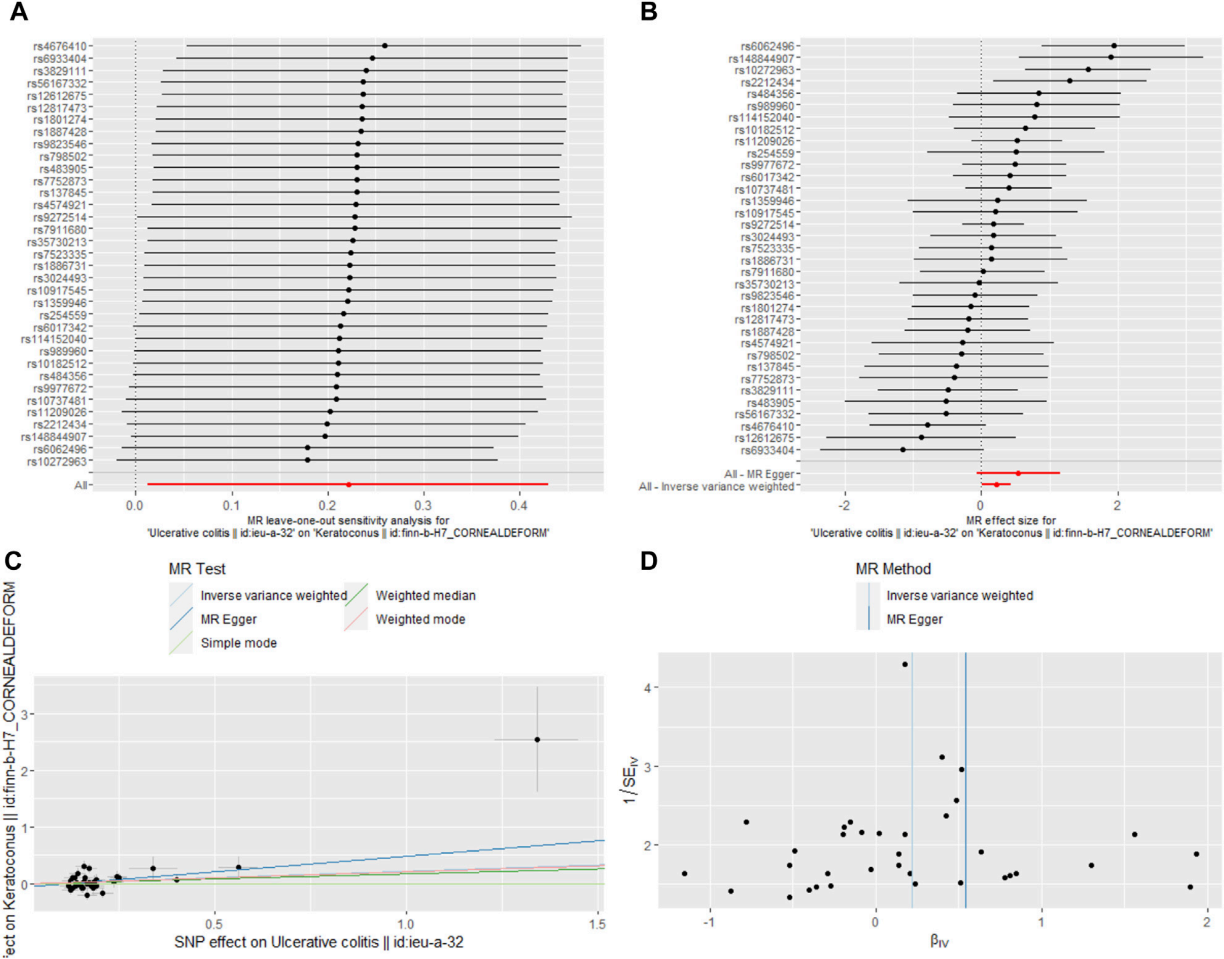
Sensitivity analysis of the causal effect of UC on keratoconus risk. **(A)** leave-one-out plot, **(B)** forest plot, **(C)** scatter plot, **(D)** funnel plot.

We also got leave-one-out plot, forest plots, test plot, scatter plot and funnel plot (Figures 2, 3). In the leave-one-out analysis, we could see that removing a single SNP did not have much effect on the overall result, and that no single SNP on the surface had a significant impact on the overall result. Through the funnel plot, we could see that the points representing causal effects were symmetrical left and right, which showed that causal effects was unlikely to be affected by potential bias.

## 4 Discussion

Keratoconus has traditionally been considered an isolated disorder that has rarely been associated with other pathological features (Krachmer et al., 1984). Eye rubbing is thought to be a risk factor, but the mechanism of action is not clear. The conjecture may be related to the presence of mechanical factors in the rubbing eye (Yang et al., 2022; Jaskiewicz et al., 2023). The accepted definition is non-inflammatory keratoectasia, but this view has recently been questioned by many studies (Galvis et al., 2015a; Taurone et al., 2021). It has been suggested that keratoconus is somewhat similar to osteoarthritis and involves a significant inflammatory process, but does not meet all criteria for inflammatory diseases. It is possible that any comorbidity that is inflammatory in nature may add synergistically to other forms of keratoconus-related inflammation and exacerbate its pathogenetic processes. It is intricate on pathophysiological process of keratoconus and inflammatory enteritis in detail. The strategy of etiological prevention plays an important role in keratoconus intervene. It is more accurate to think of keratoconus as quasi-inflammatory (Attur et al., 2002; McMonnies, 2015).

IBD is a systemic inflammatory disease of undetermined etiology and its main symptom is in the bowel, and can cause many eye complications. Ophthalmic complications are usually caused by inflammation (Ghanchi and Rembacken, 2003). Revealing the association between IBD and keratoconus may suggest the role of inflammation in keratoconus. It become an argument for the keratoconus inflammation hypothesis. Only one previous cross-sectional study and one retrospective study evaluated the causal association between IBD and keratoconus (Nemet et al., 2010; Tréchot et al., 2015). But these results are based on epidemiological data, and few studies have discussed associations from a genetic context. Our study that firstly uses mendelian randomization methods in genetic variation as instrumental variables to provide randomised evidence for assessing causal associations between IBD and keratoconus, while, it cannot replace randomized controlled trials (Ference et al., 2021). In brief, our study can support the inflammation hypothesis of keratoconus from a new angle.

We used hereditary variability as an instrumental variable to reduce the likelihood of confounding, which greatly increased the stability of the associations outcome. The causal relationship between IBD and keratoconus has been demonstrated in our study, and the mendelian randomisation also yielded consistent results, which increases the confidence in the results. However, the database we used was derived from large-scale genome-wide GWAS data, which provides strong and reliable SNPs associations and avoids potential weak instrumental bias, enabling better results and greatly improving statistical validity. Moreover, this study is firstly to use mendelian randomisation to analyse the association between IBD and keratoconus, and is filling a gap in this field.

Even though we did a lot of original study, there’s no denying, that our study has some limitations: Firstly, the samples in our study are from European origin, so whether our results can be extrapolated to other races, that needed to be verified by further data and epidemiological analyses. Second, some unclear confounding factors can bias the experimental results. In later analyses, we also obtained results of horizontal pleiotropy and heterogeneity, and the results and conclusions need to be improved. Third, IBD also includes Crohn’s disease, but our study lacked data of Crohn’s disease and did not elucidate the causal relationship between Crohn’s disease and keratoconus. While, the findings of this study have important implications for clinicians in the management of patients with IBD. Doctors may need to perform regular eye examinations for personage with IBD, in order to detect and intervene keratitis early. In addition, anti-inflammatory therapy for IBD may have potential value in preventing or slowing the progression of keratitis. Future clinical trials could explore the effects of anti-inflammatory drugs in keratitis treatment.

Mechanical injury and mechanical traction have been shown in the literature as potential pathogenic factors for keratoconus (Rabinowitz et al., 2021; Dou et al., 2022). There is also literature suggesting an association between immune factors and keratoconus (Rabinowitz et al., 2021; Dou et al., 2022). Of course, immune factors, IL-6, IL-17, also mediate the pathogenesis of IBD (Cassinotti et al., 2014; Geremia et al., 2014; Wallace et al., 2014; Saez et al., 2021; Sahoo et al., 2023). We can speculate that those related mechanical factors, cause the inflammatory response, as to leads to the onset of keratoconus. We may hypothesize that IBD and keratoconus could share certain complex immune and inflammatory responses. This implies that in patients with IBD, mechanical factors could be bypassed, and the inflammatory response could directly lead to keratoconus. Alternatively, in IBD patients, mechanical injury and traction caused by external factors, that may be more likely to provoke an inflammatory response resulted in keratoconus. Our study provides other hypotheses, such as, intestinal inflammation may lead to a systemic inflammatory response that affects distant organs, including the eyes. The specific mechanism of action needs to be further studied.

The theory of allergic reaction is one of the possible mechanisms of keratoconus, and those studies have found that keratoconus is often co-occurring with allergic diseases, such as catarrhal keratoconjunctivitis and allergic eye disease in spring. Ruedemann also reported a history of allergic reactions in most patients with keratoconus (Ruedemann, 1970; Wajnsztajn and Solomon, 2021; Seth et al., 2023). There are some similarities between keratoconus and allergic conjunctivitis (Gijs et al., 2023). Allergic reactions cause inflammation, and our study of an association between IBD and keratoconus also provides some evidence for the theory of allergic reaction in keratoconus. As for the specific link, more research is needed to further elucidate.

## 5 Conclusion

Keratoconus is an eye disease that confuses many clinical experts for the etiological factor. IBD, a systemic inflammatory disease caused by many eye complications, that is a chronic intestinal inflammatory disease of unknown cause. Mendelian randomization methods could provide randomised positively correlated evidence for assessing causal associations between IBD and keratoconus by using genetic variation as instrumental variables, and IBD was one of immediate risk factors for keratoconus.

## Data Availability

The original contributions presented in the study are included in the article/Supplementary Material, further inquiries can be directed to the corresponding author.
